# Assessment of Dietary Habits and Physical Activity Changes during the Full COVID-19 Curfew Period and Its Effect on Weight among Adults in Jeddah, Saudi Arabia

**DOI:** 10.3390/ijerph18168580

**Published:** 2021-08-13

**Authors:** Nisreen M. Abdulsalam, Najla A. Khateeb, Sarah S. Aljerbi, Waad M. Alqumayzi, Shaima S. Balubaid, Atheer A. Almarghlani, Amira A. Ayad, Leonard L. Williams

**Affiliations:** 1 Department of Food and Nutrition, Faculty of Human Sciences and Design, King Abdul Aziz University, Jeddah 21551, Saudi Arabia; nabdulsalam@kau.edu.sa (N.M.A.); Sarah_jerbi@hotmail.com (S.S.A.); waad.qumezi1@hotmail.com (W.M.A.); Shaymaaa1999@gmail.com (S.S.B.); Atheermar@gmail.com (A.A.A.); 2 Clinical Nutrition Department, College of Applied Medical Sciences, King Saud bin Abdulaziz University for Health Sciences, Al Ahsa 31982, Saudi Arabia; Khateebn@ksau-hs.edu.sa; 3 Center for Excellence in Post-Harvest Technologies, North Carolina A&T State University, 500 Laureate Way, Kannapolis, NC 28081, USA; llw@ncat.edu

**Keywords:** COVID-19, dietary habits changes, weight status, physical activity

## Abstract

The World Health Organization declared coronavirus disease 2019 (COVID-19) a pandemic in March 2020. Global efforts have been made to prevent the disease from spreading through political decisions and personal behaviors, all of which rely on public awareness. The aim of our study was to examine the effect of dietary habits on weight and physical activity (PA) during the COVID-19 stay-at-home order in Jeddah, Saudi Arabia. An online questionnaire was distributed using social media (Facebook, Twitter, Instagram, and WhatsApp) and email communication. A total of 472 adults (age range, 18–59 years), over half of the study population (68.0%) being females, 55.5% being between 19 and 29 years old, 15.0%—between 30 and 39 years old, and 11.2%—older than 50 years old, participated in the study. Our results indicated that the overall body weight was slightly increased among the 50+ age group (47.2%, *p* > 0.05), but it highly increased among the 30–39-years-old age group (32.4%, *p* > 0.05) as compared to before the pandemic lockdown period. Therefore, our results show that a significant difference (*p* < 0.05) was found for all the assessments: weight status, physical activity patterns, hours spent on screen time, homemade meals, and changes in dietary habits before and during the full COVID-19 curfew period. This study demonstrated that changes in eating habits were commonly reported among the participants who represented the full COVID-19 curfew period and that changes in eating habits and decreased physical activity led to weight gain.

## 1. Introduction

**Figure 1:**
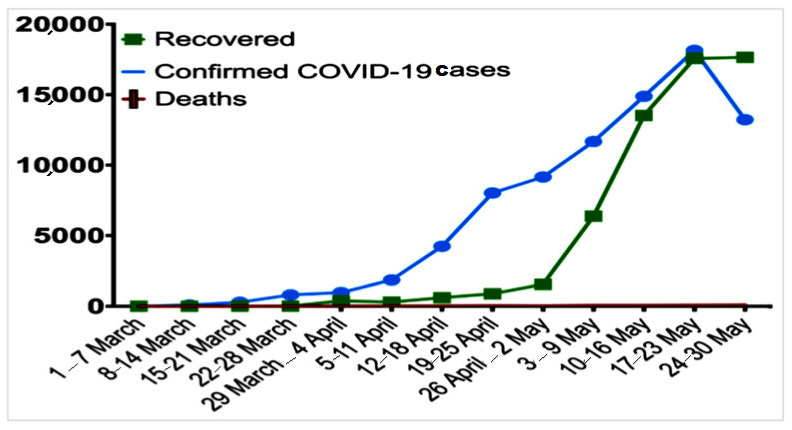
Number of confirmed deaths and recovered cases of the COVID-19 infection in Saudi Arabia from 1 March to 30 May 2020. The data are presented by weeks since the report of the first positive case in the country [6].

Coronaviruses are a large family of viruses belonging to the subfamily Orthocoronavirinae of the Coronaviridae family. Humans, bats, pigs, cats, dogs, rats, and poultry (domestic and wild animals) provide ideal habitats for virus production and metabolic activity. The disease range caused by coronaviruses in humans is considered to be more severe than that of the common cold, and the virus’s respiratory response syndrome can vary greatly between individuals. Coronaviruses, for example, can infect humans and animals and cause a variety of respiratory, enteral, liver, nephrotic, and neurological diseases [1]. Coronavirus disease 2019 (COVID-19), caused by SARS coronavirus 2 (SARS-CoV-2), is an acute respiratory syndrome considered a global situation. COVID-19 has spread and affected many countries and people. As a result of the COVID-19 outbreak, which spread rapidly to 30 million people worldwide, the World Health Organization (WHO) declared COVID-19 a global pandemic on 11 March 2020 [2]. Coronavirus has an impact on one’s health, psychological well-being, social standing, and financial situation. So far, there is no effective treatment for the COVID-19 infection, and the only way to combat this pandemic is to raise awareness and prevent infection by implementing isolation measures and improving the immune system function [3]. Many countries worldwide have closed all public gathering places, such as restaurants, shopping malls, and entertainment centers, among many others. Therefore, numerous countries have imposed complete restrictions, requiring everyone to stay at home for 24 h in order to prevent and reduce the spread of the COVID-19 disease [4]. On 6 April 2020, Saudi Arabia imposed a full curfew, prohibiting people from leaving their homes during the day and allowing them to leave only if they had an exit permit. [5]. According to the statistics of the Ministry of Health, in 2020, there were 326,930 people infected with the coronavirus only in Saudi Arabia (Figure 1) [6].

### Factors Affecting Dietary Habits

The effects of boredom on dietary habits: Boredom is a distinct emotion associated with feeling dissatisfied, restless, and unchallenged when one interprets current actions and situations as meaningless [7,8]. Several studies showed that when people are confronted with a challenge, they may attempt to escape from their sense of self-awareness and thus become completely distracted from such threats. Engaging in unhealthy behaviors that are ultimately harmful is one way to avoid this aversive self-awareness. Food, for example, helps to escape the sense of aversive self-awareness [7,9]. Boredom has been linked to a higher energy intake as well as higher fat, carbohydrate, and protein consumption. Boredom has been linked to gain weight as well as unhealthy eating behavior [10]. Eating behaviors including how much we eat, whether we eat a full meal a few times a day or have short snacks several times a day or a combination of them can be affected by external factors [11]. Therefore, continuously hearing or reading about the pandemic during quarantine can be stressful. As a result of stress, people tend to overeat, especially foods or snacks full of sugar, i.e., “comfort foods”. Studies have shown that normal-weight and overweight people report eating more when they are lonely or bored.

Association of higher screen time and dietary habits: During the current COVID-19 pandemic, the Saudi Arabia government postponed classes in all schools, colleges, and universities and suspended work, except for emergency services, in accordance with international actions and in order to combat the spread of COVID-19. As a result, it is estimated that 30,260,000 people in Saudi Arabia (89% of the population) use the Internet, 96% of the population use smartphones, and the majority of the population now have access to smartphones, laptop computers, desktop computers, and tablets to stay up-to-date with the news [12,13]. Furthermore, during the COVID-19 crisis, distance learning has been supported by technologies such as the Internet, phone, radio, TV, or phone messaging, or email communication [14]. Outcomes of numerous studies have shown less desirable snack preference and consumption and other dietary behaviors associated with extended use of smartphones among elementary school students [15,16]. Therefore, different studies have found a positive association between total screen time and body mass index (BMI) among children and adolescents. Since education and business went online, the full curfew period has led to increased screen time and bad eating habits [17,18].

The relationship between the stress level and dietary habits: In stressful and frightening situations, such as coronavirus disease 2019 (COVID-19), people tend to overeat in response to emotional triggers, which leads to more concerns and self-evaluation of body weight or shape. People are generally sociable, and this period of social isolation may have put them under psychological stress, causing them to eat more in quantity or frequency as a coping mechanism for growing fear and anxiety. Some studies indicated that the COVID-19 lockdown has been considered to influence lifestyle habits by increasing the stay-at-home time, excessive food storage, disruption of one’s routine, and heightened anxiety when hearing about the virus’s progression and spread [19,20].

The dietary habits’ impact on weight changes: Healthy eating and physical activity are key for human health and well-being, especially when the immune system is challenged [21]. Eating behavior is an important aspect of life because it can affect long-term health outcomes. Unhealthy eating habits such as skipping meals and failing to eat on time could cause a variety of health problems and nutritional deficiencies [22].

The relationship between weight, health status, and physical activity (PA): Quarantine carries some long-term effects on cardiovascular diseases, mainly related to an unhealthy lifestyle and anxiety. Following quarantine, a global action supporting healthy diets and physical activities is mandatory to encourage people to return to a healthy lifestyle routine. During quarantine, obesity made patients suffer from immense stress, making them more prone to overeating and an inactive lifestyle; this makes them prone to weight gain. Obesity is a leading risk factor for diabetes, renal disease, and cardiovascular diseases; an inflammatory state including malnutrition leads to an impaired immune response in patients suffering from obesity and susceptibility to all contagious diseases due to viruses [23]. Several studies indicated the association between body weight changes with changes in physical activity and lifestyle during an unusual situation of forced isolation or quarantine. Changes in body weight are inversely associated with changes in steps each day and mild-to-intense activity during quarantine [24,25,26].

Furthermore, the COVID-19 pandemic has affected a wide range of employees and students in terms of work and social life. Due to the lockdown, people have been forced to adapt their daily habits, including food-related behaviors which affected the weight of individuals. It is crucial to investigate how individual behavior changes over time under lockdown conditions and how crises such as the COVID-19 pandemic directly impact the behavior and health status [27]. Therefore, the aim of this study was to assess the changes in the body weight, dietary habits, and physical activity (PA) during the full COVID-19 lockdown in the City of Jeddah, Saudi Arabia.

## 2. Materials and Methods

### 2.1. Study Design and Participants

This cross-sectional study was conducted on a sample of the population in Jeddah, Saudi Arabia, during the full COVID-19 curfew period. The survey was designed following the protocol as per Reyes-Olavarría et al. [25] with slight modifications and uploaded to the Google Forms platform. The developed questionnaire (originally written in English) was translated into Arabic and then translated back into English. The survey was tested and validated by experts at the food and nutrition department at King Abdul Aziz University, SA, to ensure that the questions were appropriate and avoided any misunderstanding in order to answer them in the intended way. Online questionnaires were distributed via social media (Facebook, Twitter, Instagram, and WhatsApp) and emails communication. The target population consisted of both men and women aged 18–59 years who had experienced the full COVID-19 curfew period. We received 531 answers; after excluding the answers that met the exclusion criteria, such as living in a different region, incomplete survey, out of the age range, and lack of any data, the final dataset included 472 participants.

#### 2.1.1. Ethical Aspects of This Research

At the start of the survey, study information was briefly presented. The poll was completely anonymous, no sensitive information was collected, and the participants had the option to leave at any point if they felt uncomfortable. The research was carried out in accordance with the Declaration of Helsinki’s criteria. According to S. Gupta (2017), consent is considered knowledgeable when participants understand the purpose of the collection of information about them as well as their right to give, withhold, or withdraw consent at any moment [28].

#### 2.1.2. Demographic Information

The demographic part consisted of such collected personal information as age, gender, education level, socioeconomic status, place of residence, marital status, and professional situation during the pandemic.

#### 2.1.3. Weight Status and Physical Activity Patterns

This section was associated with the participants’ weight status and their physical activity patterns. They were asked for information on body weight before and after the full COVID-19 lockdown, as well as the percentage of body weight change during the full curfew period. We asked about the frequency of physical activity each week and the number of hours and minutes allocated to physical activity. The participants were asked if they had checked social networks to find exercise routines and about the amount of time, they had spent watching television or social media defined as screen time.

#### 2.1.4. Food Habits

For the set of questions associated with the participants’ eating habits, questions on weekly and daily food intake patterns were asked. In addition, questions pertaining to the overall increase, maintenance, or decrease in dietary intake, and whether the participants cooked more, less, or as much as before the full COVID-19 curfew period. The general dietary experience was evaluated and compared to the time before the full COVID-19 curfew period.

### 2.2. Statistical Analysis

Data analysis was conducted using IBM SPSS (Statistical Package for Social Sciences) (IBM Corp. Released 2017. IBM SPSS Statistics for Windows, Version 25.0. Armonk, NY, USA: IBM Corp.). All the variables were analyzed qualitatively and were expressed as the percentages (%) and numbers (*n*). Descriptive statistics were presented as frequencies and percentages. The Kruskal–Wallis test and the Wilcoxon rank sum test are rank sum test extended to more than two samples. Weight status, physical activity patterns, and dietary habits were the variables used in the tests to determine whether there was a significant difference for all the seven assessments. The main outcomes were unhealthy dietary and lifestyle changes described as decreased physical activity (vs. the same or increased), increased weight (vs. the same or decreased) and increased food intake (vs. the same or decreased); *p* < 0.05 was considered statistically significant.

## 3. Results

### Sociodemographic Characteristics

Data on age, gender, education level, place of residence, and professional situation during the pandemic were collected. Table 1 describes the sociodemographic characteristics of the study population. More than a half of the study population (68.0%) were females, 55.5% were between 19 and 29 years old, 15.0%—between 30 and 39 years old, and 11.2% were older than 50 years old. Half of the study population (50.0%) were unmarried, and the largest professional group consisted of undergraduate students (39.6%). Most of the participants (53.2%) were from middle-income families with the monthly family income for the past 6 months of up to SAR 10,000 (US$ 2600). Moreover, about 84.5% of the participants lived in the Western district.

Furthermore, the comparison of the weight status, physical activity patterns, and dietary habits (before and during the full COVID-19 curfew period) is shown in Table 2. The proportion of respondents who were overweight slightly increased from 32.8% before the COVID-19 period to 38.8% during the COVID-19 period (*p* < 0.05). Our results showed that in 27.3% of the participants, body weight increased by 2–4 kg during the COVID-19 period (*p* > 0.05). Before the COVID-19 pandemic, 12.5% of the respondents spent more than 6 hours/day using computers/mobile devices, while during the COVID-19 pandemic, the category of the participants spending more than 6 h/day in front of the computer and/or phone and/or TV screen increased significantly to become the most prevalent (36.2%; *p* < 0.05). In addition to that, the normal daily activity level significantly decreased (*p* < 0.05) during the COVID-19 period. Furthermore, our results indicate that there were no significant changes (*p* > 0.05) in the overall habits of eating healthy food before and during the COVID-19 pandemic. More than half of the respondents (56.4%) ate homemade meals during the COVID-19 period, which was significantly higher than before the pandemic. Therefore, takeaway and ordering from restaurants significantly decreased (*p* < 0.05) from 64.0% to 35.4% during the COVID-19 pandemic. However, daily late-night snacking was significantly (*p* < 0.05) more frequent during the COVID-19 period, with the increase from 8.7% to 16.5%.

Moreover, the respondents’ body weight status during the full COVID-19 curfew period is presented in Table 3; 41.7% of the participants were males, and their body weight slightly increased during the pandemic. The results showed that there were no significant differences between males and females regarding the body weight status (*p* > 0.05).

The data in Table 4 show the overall body weight after the pandemic lockdown slightly increased in the 50+ age group (47.2%, *p* > 0.05) and highly increased in the 30–39-years-old age group (32.4%, *p* > 0.05) compared to before the pandemic lockdown period. Therefore, there were no significant differences between age groups (*p* > 0.05). Furthermore, there was a slight increase (57.95%, *p* > 0.05) in the body weight in the low-income participants and a high increase (25.5%, *p* > 0.05) in the body weight in the middle-income participants (see Table 5).

The data in Table 6 present the body weight status during the full COVID-19 curfew period and its relation to the participants’ education level. There were no statistically significant differences between the education level and the body weight (*p* > 0.05). Most of the participants (42.9%, *p* < 0.05) spent 5–6 h in front of the computer/phone/TV screen; their body weight slightly increased during the full COVID-19 stay-at-home period. Therefore, there are significant differences between the number of hours spent in front of the computer/phone/TV and the changes in the body weight status (*p* < 0.05) (Table 7).

Most of the participants (39.8%, *p* < 0.05) did not practice any physical activity when they spent more than 6 h/day using digital media such as desktops, laptops, TV, and phones. The results in Table 8 indicate that there are statistically significant differences between the number of hours spent in front of the computer/phone/TV and physical activity (*p* < 0.05) during the full COVID-19 curfew period. Additionally, most of the participants (82.4%, *p* < 0.05) preferred to eat homemade meals during the full COVID-19 curfew period which was significantly more prevalent than ordering from restaurants or takeaway (Table 9).

## 4. Discussion

Our research sought to identify changes in lifestyle such as food habits and PA patterns in the City of Jeddah, Saudi Arabia, during the COVID-19 confinement period and examine their relationship with changes in body weight. Moreover, eating habits and physical activity during the full COVID-19 curfew period in Jeddah, Saudi Arabia, was impacted by food inaccessibility, availability of goods, and limited market hours. Our results indicated that the COVID-19 pandemic had a negative impact on the participants’ physical activity patterns. For instance, the number of people practicing PA at least 4 h/week before the COVID-19 pandemic decreased from 8.7% to 5.3%, while the number of people who did not do any physical activity increased from 31.6% to 35.2%. As a result, the number of participants with higher body weight increased from 33% to 38.8%, which is consistent with the findings of other authors [17,24,29]. Additionally, our results also agree with the work of Haddad et al. (2020) who found a significant prevalence of lower physical activity among Lebanese adults, whereby 41% reported no physical activity or exercise during lockdown [29]. Other studies have indicated that reducing physical activity leads to the loss of muscle mass in a matter of days [30] and that metabolic consequences of a prolonged sedentary lifestyle such as increased body weight, including fat mass, and impaired glycemic control are observed in healthy adults [31].

Furthermore, our study reports that the overall poor eating habits slightly increased in prevalence from 17.6% to 27.3% during the pandemic. Our results showed that the number of participants spending more than 6 h daily in front of the TV/laptop/phones significantly increased from 12.5% to 36.2%. Our results are consistent with other studies that staying at home for an extended period of time such as the COVID-19 pandemic may result in an increase in screen-based activities (video games, TV watching, use of mobile devices) which increases the body weight [32,33].

In line with previous research, which found an increase in homemade meal consumption and a decrease in fast food consumption [34], our results also showed a significant increase in the consumption of homemade meals (from 26.9% to 56.4%), while the prevalence of ordering from restaurants, takeaway, and deliveries decreased from 64% to 35.4%. However, other studies indicated that limited access to daily grocery shopping resulted in the decrease in consumption of fresh foods in favor of highly processed ones, such as convenience foods, junk foods, snacks, and ready-to-eat cereals, which are high in fats, sugars, and salt [35,36]. Our results showed that during the COVID-19 lockdown, people spent more time watching TV / using a laptop/phone which is associated with snacking frequency, especially late-night snacking, and fast foods.

### Limitations of the Study

Although this study provides an insight into how a pandemic-related lockdown can affect dietary patterns and weight, there are some limitations that also need to be underlined. Firstly, the research on our topic needs a longer period and a larger sample size to provide more accurate and better results. Secondly, the survey sample could be distributed over more regions and cities to clarify the differences and points of conflict. Thirdly, adding a comparison between gender and physical activity would clarify the effect of the full COVID-19 curfew period on the physical activity for groups whose physical activity percentage was high, which was the reason for the difference between weight gain for males and females. Fourthly, measuring the fat rate and the BMI in determining the percentage change in weight and the 24-h recall or any of the parameters of a person’s nutritional status would help get better results. Lastly, a question about the food types, portion sizes, food ingredients, and even the cooking process could be included into the future questionnaire and taken into consideration. This may improve the results regarding the overall dietary habits. However, to the best of our knowledge, this is the first study to document preliminary dietary changes in the City of Jeddah, Saudi Arabia, during the COVID-19 epidemic, and therefore it can provide early background information on the topic.

## 5. Conclusions

This study demonstrated that changes in eating habits were commonly reported among participants who represented the full COVID-19 curfew period and that changes in eating habits and decreased physical activity led to weight gain. Most of the population showed an increase in weight, an increase in food consumption, and a decrease in physical activity. According to our findings, people who gained weight started eating more, especially late-night snacks or meals, spent more time in front of the TV and/or laptop screen, and engaged in less physical exercise. Therefore, the responsible authorities should focus on public awareness initiatives to reduce the long-term effects of the full COVID-19 curfew on people’s eating patterns and general food safety in Saudi Arabia. In addition, encouraging healthy eating habits and attitudes as well as increasing physical activity can help to enhance body health and prevent weight gain.

## Figures and Tables

**Table 1:** Sociodemographic characteristics of the participants (*n* = 472).

Measure		Frequency (*n*)	Percentage (%)
Gender			
Female		321	68.0*
Male		151	32.0
Age (Years)			
18 and under		41	8.7
19–29		262	55.5*
30–39		71	15.0
40–49		45	9.5
50 and over		53	11.2
Marital status			
Married		197	41.7
Divorced		21	4.4
Separated		12	2.5
Widowed		6	1.3
Unmarried		236	50.0*
Level of the total family income for the past 6 months			
Low		19	4.0
Middle-low		77	16.3
Middle		251	53.2*
Middle-high		94	19.9
High		31	6.6
Family income (monthly)			
Saudi Riyals (SR)	US dollars ($)		
Less than 1000	Less than 266.6	16	3.4
1001–5000	266–1333.2	102	21.6
5001–10,000	1333–2666	104	22.0*
10,001–15,000	2666–4000	73	15.5
15,001–20,000	Less than 5000	79	16.7
20,001–25,000	Less than 7000	50	10.6
25,001 and over	Over 7000	48	10.2
How many family members live in the same house?			
1 person		15	3.2
2–4 persons		172	36.4*
5–6 persons		166	35.2
7–9 persons		111	23.5
10 persons or more		8	1.7
What is your current employment status?			
Full-time employment		124	26.3
Part-time employment		43	9.1
Unemployed		15	3.2
Self-employed		13	2.8
Homemaker		63	13.3
Student		187	39.6*
Retired		27	5.7
What is your highest level of education?			
Less than high school		16	3.4
High school		102	21.6
Some college		81	17.2
Bachelor’s degree		199	42.2*
Master’s degree		38	8.1
Doctoral degree		35	7.4
Professional degree		1	0.2
Region of the country			
Middle region		15	3.2
Northern region		24	5.1
Eastern region		10	2.1
Western region		399	84.5 *
South region		24	5.1
Do you live in the City of Jeddah currently?			
Yes		472	100.0
No		0	0.0

* Indicates the most prevalent group.

**Table 2:** Comparison of the body weight, physical activity (PA), and dietary habits before and during the COVID-19 lockdown period.

Weight and Physical Activity Patterns					
	Before the COVID-19 Pandemic (*n* = 472)		During the COVID-19 Pandemic (*n* = 472)		*p*-Value
Body weight status	*n*	(%)	*n*	(%)	0.002 ^a^
Slightly increased	155	32.8	183	38.8	
Highly increased	58	12.3	111	23.5	
Nether increased nor decreased	237	50.2	130	27.5	
Slightly decreased	18	3.8	29	6.1	
Highly decreased	4	0.8	19	4.0	
Absolute body weight change	*n*	(%)	*n*	(%)	0.345 ^a^
Increased by less than 1 kg	151	32.0	90	19.1	
Increased by 2–4 kg	107	22.7	129	27.3	
Increased by more than 5 kg	55	11.7	114	24.2	
Decreased by less than 1 kg	34	7.2	29	6.1	
Decreased by 2–4 kg	16	3.4	38	8.1	
Decrease by more than 5 kg	5	1.1	21	4.4	
No change	104	22.0	51	10.8	
Hours spent in front of the computer/mobile devices/TV	*n*	(%)	*n*	(%)	0.000 ^a^
Less than one hour a day	43	9.1	22	4.7	
1–2 h per day	114	24.2	36	7.6	
3–4 h per day	156	33.1	96	20.3	
5–6 h per day	100	21.2	147	31.1	
More than 6 h per day	59	12.5	171	36.2	
Physical activity	*n*	(%)	*n*	(%)	0.000 ^a^
I do not practice any physical activity	149	31.6	166	35.2	
Less than 1 h per week	102	21.6	114	24.2	
1–2 h per week	100	21.2	111	23.5	
3–4 h per week	80	16.9	56	11.9	
More than 4 h per week	41	8.7	25	5.3	
Activity level per day	*n*	(%)	*n*	(%)	0.000 ^a^
Low	82	17.4	137	29.0	
Very low	55	11.7	105	22.2	
Normal	243	51.5	164	34.7	
High	84	17.8	62	13.1	
Very high	8	1.7	4	0.8	
Dietary habits					
	Before the COVID-19 pandemic (*n* = 472)		During the COVID-19 pandemic (*n* = 472)		*p*-value
Overall habits of eating healthy food	*n*	(%)	*n*	(%)	0.865 ^a^
Poor	83	17.6	129	27.3	
Fair	215	45.6	144	30.5	
Good	125	26.5	128	27.1	
Very good	39	8.3	54	11.4	
Excellent	10	2.1	17	3.6	
Homemade meals per week	*n*	(%)	*n*	(%)	0.000 ^a^
0 times/week	28	5.9	30	6.4	
1–2 times/week	118	25.0	61	12.9	
3–6 times/week	199	42.2	115	24.4	
Daily	127	26.9	266	56.4	
Ordering from a restaurant, takeaway, and deliveries per week	*n*	(%)	*n*	(%)	0.000 ^a^
0 times/week	60	12.7	205	43.4	
1–2 times/week	302	64.0	167	35.4	
3–6 times/week	94	19.9	72	15.3	
Daily	16	3.4	28	5.9	
Late-night snack or meal	*n*	(%)	*n*	(%)	0.000 ^a^
Never	73	15.5	45	9.5	
Rarely	129	27.3	76	16.1	
Occasionally	163	34.5	143	30.3	
Usually	66	14.0	130	27.5	
Always	41	8.7	78	16.5	

^a^ Wilcoxon signed-rank test.

**Table 3:** Comparison of the body weight status and gender during the full COVID-19 curfew period.

Body Weight Status	Gender				*p*-Value
	Male		Female		
	*n*	(%)	*n*	(%)	
Slightly increased	63	41.7	120	37.4	0.074 ^a^
Highly increased	39	25.8	72	22.4	
Nether increased nor decreased	43	28.5	87	27.1	
Slightly decreased	5	3.3	24	7.5	
Highly decreased	1	0.7	18	5.6	

^a^ Kruskal–Wallis test.

**Table 4:** Comparison of the body weight status and age during the full COVID-19 curfew period.

Body Weight Status	Age (years)										*p*-Value
	Under 18		19–29		30–39		40–49		50 and Over		
	*n*	(%)	*n*	(%)	*n*	(%)	*n*	(%)	*n*	(%)	
Slightly increased	11	26.8	107	40.8	23	32.4	17	37.8	25	47.2	0.098 ^a^
Highly increased	12	29.3	50	19.1	23	32.4	11	24.4	15	28.3	
Nether increased nor decreased	11	26.8	74	28.2	21	29.6	13	28.9	11	20.8	
Slightly decreased	3	7.3	18	6.9	4	5.6	2	4.4	2	3.8	
Highly decreased	4	9.8	13	5.0	0	0.0	2	4.4	0	0.0	

^a^ Kruskal–Wallis test.

**Table 5:** Comparison of the body weight status and the family income for the past 6 months during the pandemic.

Body Weight Status	Level of the Total Family Income for the Past 6 Months										
	Low		Middle-Low		Middle		Middle-High		High		*p*-Value
	*n*	(%)	*n*	(%)	*n*	(%)	*n*	(%)	*n*	(%)	
Slightly increased	11	57.9	29	37.7	96	38.2	37	39.4	10	32.3	0.225 ^a^
Highly increased	2	10.5	11	14.3	64	25.5	25	26.6	9	29.0	
Neither increased nor decreased	6	31.6	28	36.4	60	23.9	26	27.7	10	32.3	
Slightly decreased	0	0.0	6	7.8	20	8.0	2	2.1	1	3.2	
Highly decreased	0	0.0	3	3.9	11	4.4	4	4.3	1	3.2	

^a^ Kruskal–Wallis test.

**Table 6:** Association of the body weight status and the highest level of education during the full COVID-19 pandemic curfew period.

Body Weight Status	Highest Level of Education														
	Less Than High School		High School		Some College		Bachelor’s Degree		Master’s Degree		Doctoral Degree		Professional Degree		*p*-Value
	*n*	(%)	*n*	(%)	*n*	(%)	*n*	(%)	*n*	(%)	*n*	(%)	*n*	(%)	
Slightly increased	5	31.3	41	40.2	29	35.8	84	42.2	12	31.6	12	34.3	0	0.0	0.645 ^a^
Highly increased	4	25.0	20	19.6	20	24.7	45	22.6	12	31.6	9	25.7	1	100	
Neither increased nor decreased	7	43.8	28	27.5	21	25.9	50	25.1	13	34.2	11	31.4	0	0.0	
Slightly decreased	0	0.0	6	5.9	6	7.4	15	7.5	1	2.6	1	2.9	0	0.0	
Highly decreased	0	0.0	7	6.9	5	6.2	5	2.5	0	0.0	2	5.7	0	0.0	

^a^ Kruskal–Wallis test.

**Table 7:** Comparison of the body weight status and the hours spent in front of the computer/mobile devices/TV during the full COVID-19 curfew period.

Body Weight Status	Time Spent in front of the Computer/Mobile Devices/TV (Hours/Day)										
	Less Than 1		1–2		3–4		5–6		More Than 6		*p*-Value
	*n*	(%)	*n*	(%)	*n*	(%)	*n*	(%)	*n*	(%)	
Slightly increased	5	22.7	16	44.4	41	42.7	63	42.9	58	33.9	0.003 ^a^
Highly increased	0	0.0	4	11.1	26	27.1	37	25.2	44	25.7	
Neither increased nor decreased	17	77.3	14	38.9	25	26.0	34	23.1	40	23.4	
Slightly decreased	0	0	2	5.6	3	3.1	9	6.1	15	8.8	
Highly decreased	0	0	0	0	1	1.0	4	2.7	14	8.2	

^a^ Kruskal–Wallis test.

**Table 8:** Comparison of the physical activity (PA) and the hours spent in front of the computer/mobile devices/TV during the full COVID-19 curfew period.

	Time Spent in front of the Computer/Mobile Devices/TV (Hours/Day)										
	Less Than 1		1–2		3–4		5–6		More Than 6		*p*-Value
	*n*	(%)	*n*	(%)	*n*	(%)	*n*	(%)	*n*	(%)	
I do not practice any physical activity	15	68.2	10	27.8	27	28.1	46	31.3	68	39.8	0.004 ^a^
Less than 1 h per week	4	18.2	15	41.7	24	25.0	30	20.4	41	24.0	
1–2 h per week	1	4.5	10	27.8	27	28.1	41	27.9	32	18.7	
3–4 h per week	2	9.1	1	2.8	13	13.5	19	12.9	21	12.3	
More than 4 h per week	0	0.0	0	0.0	5	5.2	11	7.5	9	5.3	

^a^ Kruskal–Wallis test.

**Table 9:** Comparison of the homemade meals per week and ordering from a restaurant, takeaway, and deliveries per week during the full COVID-19 curfew period.

Ordering from a Restaurant, Takeaway, and Deliveries per Week (Times/Week)									
	0		1–2		3–6		Daily		*p*-Value
Homemade Meals per Week	*n*	(%)	*n*	(%)	*n*	(%)	*n*	(%)	0.000 ^a^
0 times/week	9	4.4	5	3.0	8	11.1	8	28.6	
1–2 times/week	7	3.4	26	15.6	22	30.6	6	21.4	
3–6 times/week	20	9.8	65	38.9	25	34.7	5	17.9	
Daily	169	82.4	71	42.5	17	23.6	9	32.1	

^a^ Kruskal–Wallis test.

## References

[1] Ghosh S., Malik Y.S. (2020). Drawing Comparisons between SARS-CoV-2 and the Animal Coronaviruses. Microorganisms.

[2] World Health Organization (2020). WHO Director-General’s Remarks at the Media Briefing on 2019-nCoV on 11 February 2020. https://www.who.int/director-general/speeches/detail/who-director-general-s-remarks-at-the-media-briefing-on-2019-ncov-on-11-february-2020.

[3] CDC (2017). Quarantine and Isolation Quarantine. https://www.cdc.gov/quarantine/quarantineisolation.html.

[4] Ruiz-Roso M.B., Knott-Torcal C., Matilla-Escalante D.C., Garcimartín A., Sampedro-Nuñez M.A., Dávalos A., Marazuela M. (2020). COVID-19 lockdown and changes of the dietary pattern and physical activity habits in a cohort of patients with type 2 diabetes mellitus. Nutrients.

[5] Ministry of Interior (2020). Ministry of Interior. https://www.moi.gov.sa/wps/portal/Home/Home/!ut/p/z1/04_Sj9CPykssy0xPLMnMz0vMAfIjo8ziLQPdnT08TIy83Q0dzQwcPc2N_A08TQ3dPY30wwkpiAJKG-AAjgZA_VFgJc7ujh4m5j4GBhY-7qYGno4eoUGWgcbGBo7GUAV4zCjIjTDIdFRUBAApuVo7/dz/d5/L0lDUmlTUSEhL3dHa0FKRnNBLzROV3FpQSEhL2Fy/.

[6] Obied D.A., Alhamlan F.S., Al-Qahtani A.A., Al-Ahdal M.N. (2020). Containment of COVID-19: The unprecedented response of Saudi Arabia. JIDC.

[7] Van Tilburg W.A., Igou E.R. (2012). On boredom: Lack of challenge and meaning as distinct boredom experiences. Motiv. Emot..

[8] Bozaci I. (2020). The effect of boredom proneness on smartphone addiction and impulse purchasing: A field study with young consumers in turkey. JAFEB.

[9] Moynihan A.B., Van Tilburg W.A., Igou E.R., Wisman A., Donnelly A.E., Mulcaire J.B. (2015). Eaten up by boredom: Consuming food to escape awareness of the bored self. Front. Psychol..

[10] Muscogiuri G., Barrea L., Savastano S., Colao A. (2020). Nutritional recommendations for CoVID-19 quarantine. Eur. J. Clin. Nutr..

[11] Alzahrani S.H., Saeedi A.A., Baamer M.K., Shalabi A.F., Alzahrani A.M. (2020). Eating habits among medical students at King Abdulaziz University, Jeddah, Saudi Arabia. Int. J. Gen. Med..

[12] Hassounah M., Raheel H., Alhefzi M. (2020). Digital response during the COVID-19 pandemic in Saudi Arabia. J. Med. Internet Res..

[13] Kim H., Pae M. (2017). Lifestyle, dietary behavior and snack preference of upper-grade elementary school students in Cheongju according to the usage time of smartphones. KJCN.

[14] Basilaia G., Kvavadze D. (2020). Transition to online education in schools during a SARS-CoV-2 coronavirus (COVID-19) pandemic in Georgia. PEDRE.

[15] Khan M.A., Smith J.E.M. (2020). “Covibesity,” a new pandemic. Obes. Med..

[16] Kaur K. (2019). FOOD HABITS OF SCHOOL GOING CHILDREN: INFLUENCE OF TELEVISION ADVERTISEMENT. Br. Food J..

[17] Ammar A., Brach M., Trabelsi K., Chtourou H., Boukhris O., Masmoudi L., Bouaziz B., Bentlage E., How D., Ahmed M. (2020). Effects of COVID-19 home confinement on eating behaviour and physical activity: Results of the ECLB-COVID19 international online survey. Nutrients.

[18] Pokhrel S., Chhetri R. (2021). A literature review on impact of COVID-19 pandemic on teaching and learning. High. Educ. Future.

[19] Park S.H., Lee E.J., Chang K.J. (2020). Dietary habits and snack consumption behaviors according to level of job stress among 20-to 30-year old office workers in the Seoul metropolitan area. J. Korean Soc. Food Cult..

[20] Di Renzo L., Gualtieri P., Pivari F., Soldati L., Attinà A., Cinelli G., Leggeri C., Caparello G., Barrea L., Scerbo F. (2020). Eating habits and lifestyle changes during COVID-19 lockdown: An Italian survey. J. Transl. Med..

[21] Calder P.C. (2020). Nutrition, immunity and COVID-19. BMJ Nutr. Prev. Health.

[22] Stephenson J., Heslehurst N., Hall J., Schoenaker D.A., Hutchinson J., Cade J.E., Poston L., Barrett G., Crozier S.R., Barker M. (2018). Before the beginning: Nutrition and lifestyle in the preconception period and its importance for future health. Lancet.

[23] Mattioli A.V., Sciomer S., Cocchi C., Maffei S., Gallina S. (2020). Quarantine during COVID-19 outbreak: Changes in diet and physical activity increase the risk of cardiovascular disease. Nutr. Metab. Cardiovasc. Dis..

[24] He M., Xian Y., Lv X., He J., Ren Y. (2020). Changes in body weight, physical activity, and lifestyle during the semi-lockdown period after the outbreak of COVID-19 in China: An online survey. Disaster. Med. Public Health Prep..

[25] Reyes-Olavarría D., Latorre-Román P.Á., Guzmán-Guzmán I.P., Jerez-Mayorga D., Caamaño-Navarrete F., Delgado-Floody P. (2020). Positive and negative changes in food habits, physical activity patterns, and weight status during COVID-19 confinement: Associated factors in the Chilean population. Int. J. Environ. Res. Public Health.

[26] Husain W., Ashkanani F. (2020). Does COVID-19 change dietary habits and lifestyle behaviours in Kuwait: A community-based cross-sectional study. Environ. Health Prev. Med..

[27] Zhong B., Huang Y., Liu Q. (2021). Mental health toll from the coronavirus: Social media usage reveals Wuhan residents’ depression and secondary trauma in the COVID-19 outbreak. Comput. Hum. Behav..

[28] Gupta S. (2017). Ethical issues in designing internet-based research: Recommendations for good practice. JRP.

[29] Haddad C., Zakhour M., Haddad R., Al Hachach M., Sacre H., Salameh P. (2020). Association between eating behavior and quarantine/confinement stressors during the coronavirus disease 2019 outbreak. J. Eat. Disord..

[30] Westerterp K.R. (2019). Physical Activity and Body-Weight Regulation.

[31] Jakicic J.M., Davis K.K. (2011). Obesity and physical activity. Psychiatr. Clin. N. Am..

[32] Mason T.B., Barrington-Trimis J., Leventhal A.M. (2021). Eating to cope with the COVID-19 pandemic and body weight change in young adults. J. Adolesc. Health.

[33] AlMughamis N., AlAsfour S., Mehmood S. (2020). Poor eating habits and predictors of weight gain during the COVID-19 quarantine measures in Kuwait: A cross sectional study. F1000Research.

[34] Górnicka M., Drywień M.E., Zielinska M.A., Hamułka J. (2020). Dietary and lifestyle changes during COVID-19 and the subsequent lockdowns among Polish adults: A cross-sectional online survey PLifeCOVID-19 study. Nutrients.

[35] Zupo R., Castellana F., Sardone R., Sila A., Giagulli V.A., Triggiani V., Cincione R.I., Giannelli G., De Pergola G. (2020). Preliminary trajectories in dietary behaviors during the COVID-19 pandemic: A public health call to action to face obesity. Int. J. Environ. Res. Public Health.

[36] Scarmozzino F., Visioli F. (2020). Covid-19 and the subsequent lockdown modified dietary habits of almost half the population in an Italian sample. Foods.

